# Subnetwork representation learning for discovering network biomarkers in predicting lymph node metastasis in early oral cancer

**DOI:** 10.1038/s41598-021-03333-5

**Published:** 2021-12-14

**Authors:** Minsu Kim, Sangseon Lee, Sangsoo Lim, Doh Young Lee, Sun Kim

**Affiliations:** 1 Computer Science and Mathematics Division, Oak Ridge National Laboratory, Oak Ridge, TN 37831 USA; 2 Institute of Computer Technology, Seoul National University, Seoul, 08826 Korea; 3 Bioinformatics Institute, Seoul National University, Seoul, 08826 Korea; 4 Seoul National University College of Medicine, Seoul, 03080 Korea; 5 Department of Computer Science and Engineering, Seoul National University, Seoul, 08826 Korea; 6 Interdisciplinary Program in Bioinformatics, Seoul National University, Seoul, 08826 Korea; 7 Interdisciplinary Program in Artificial Intelligence, Seoul National University, Seoul, 08826 Korea; 8 AIgenDrug, Co., Ltd, Seoul, Korea

**Keywords:** Transcriptomics, Diagnostic markers

## Abstract

Cervical lymph node metastasis is the leading cause of poor prognosis in oral tongue squamous cell carcinoma and also occurs in the early stages. The current clinical diagnosis depends on a physical examination that is not enough to determine whether micrometastasis remains. The transcriptome profiling technique has shown great potential for predicting micrometastasis by capturing the dynamic activation state of genes. However, there are several technical challenges in using transcriptome data to model patient conditions: (1) An Insufficient number of samples compared to the number of genes, (2) Complex dependence between genes that govern the cancer phenotype, and (3) Heterogeneity between patients between cohorts that differ geographically and racially. We developed a computational framework to learn the subnetwork representation of the transcriptome to discover network biomarkers and determine the potential of metastasis in early oral tongue squamous cell carcinoma. Our method achieved high accuracy in predicting the potential of metastasis in two geographically and racially different groups of patients. The robustness of the model and the reproducibility of the discovered network biomarkers show great potential as a tool to diagnose lymph node metastasis in early oral cancer.

## Introduction

Oral tongue squamous cell carcinoma (OTSCC) is one of the most common malignant tumors in the oral cavity^1^. Cervical lymph node metastasis is a major factor in a poor prognosis for OTSCC and also occurs even in early stages^2^. Currently, clinical diagnosis relies on physical examinations such as palpation, ultrasonography, computed tomography (CT-scan), and magnetic resonance imaging (MRI). Unfortunately, these physical examinations are not accurate enough to determine if micrometastasis remains in the lesion. Micrometastasis indicates that a small number of cancer cells that have spread from the primary tumor to other parts of the body are too few to be detected by screening or physical examination. For this reason, clinicians recommend lymphadenectomy for patients who do not require resection^3^. Lymphadenectomy refers to surgery to remove lymph nodes, which can cause serious side effects. Therefore, being able to detect micrometastases with molecular-level data could be of significant benefit to patients with OTSCC.

Transcriptome data are whole genome-scale molecular profiles generated by high-throughput RNA profiling techniques such as microarrays and RNA sequencing (RNA-seq), which are known to have great potential to identify micrometastasis in cancer patients^4–6^. There are several challenges in modeling patient conditions using transcriptome data. First, despite advances in high-throughput RNA profiling technology, the cost of production per sample is still at a non-negligible level, and the number of genes to consider is relatively large compared to the number of samples, which is a challenge for many researchers. This problem is also referred to as the low sample high dimension problem^7^. In addition, cellular proteins rarely act individually and generally cooperate to perform specific functions and express a specific phenotype^8^. Therefore, the complex dependence between genes due to protein interactions should also be considered. Finally, heterogeneity between patient samples is known to have a significant impact on cohort studies due to the genetic diversity between individuals with different geographic and ethnic backgrounds^9, 10^.

Subnetwork level representation (SLR) is one of the most promising ways to reduce the high dimensions of transcriptome data using biological networks. Studies have shown that the SLR approach using protein-protein interaction (PPI) network is excellent for predicting the clinical status of cancer patients in terms of robustness and effectiveness^11–14^. Additionally, the SLR approach can provide a comprehensive understanding of the underlying mechanisms by which the disease progresses and influences prognosis^15, 16^.

The biggest challenge when using PPI networks is the sparse network representation^17, 18^. The integration of the gene expression matrix and the adjacent matrix is not an easy task even without sparsity because they have completely different shapes. Subsystem Activation Score (SAS) is one of the most effective ways to solve this problem^19^. SAS introduced a natural way to integrate PPI networks with the transcriptome. In a recent study, Lim et al.^20^ compared several SLR methods including SAS in terms of 1) reproducibility of RNA-seq data characteristics, 2) robustness to noise, 3) classification for tumor versus normal information, 4) classification for survival information, and 5) classification for cancer subtypes. They devised various statistics to measure the performance of each method on each criterion. The study showed that SAS has the best overall performance compared to other SRL methods when evaluated for the above five criteria.

According to BioGrid^21^ , each gene has an average number of PPI interactions of 9.56, meaning each gene is linked to an average of 9.56 genes. Since there are at least 20 000 whole genomes, looking at the vector representation of the adjacency matrix of the PPI network, these vectors will look very sparse. That is, most values are 0 and very few (average 9.56) values are 1. As Perozzi et al.^18^ stated, this sparsity can make generalizations in statistical learning models extremely difficult. DeepWalk^18^ is a well-designed solution to this sparsity problem and we wanted to take advantage of it. The proposed method was largely motivated by the work of Perozzi et al.^18^ using DeepWalk as a graph embedding method.

In this paper, we propose a method to discover network biomarkers and determine their metastasis potential in early OTSCCs designed to overcome the aforementioned challenges. To achieve this goal, we have developed three new computational techniques that are combined into a single computational framework, including a supervised subnetwork level representation learning system for extending SAS , a subnetwork extraction method using the DeepWalk graph embedding technique , and an attention-based classification system for integrating subnetwork level representations and discovering network biomarkers. In the following sections, we described (1) how the proposed method defined subnetworks for identifying network biomarkers, (2) how the problem is addressed as a machine-learning framework that calculated the representation of each subnetwork based on the given input and target variables, and (3) how it was applied to early oral cancer to predict lymph node metastasis.

## Materials and methods

The proposed method works in three stages: (1) subnetwork extraction using graph embedding technique, (2) construction of subnetwork level representation, and (3) integration of subnetwork level representation into the master-level decision.

### Subnetwork extraction using graph embedding technique

**Figure 1:**
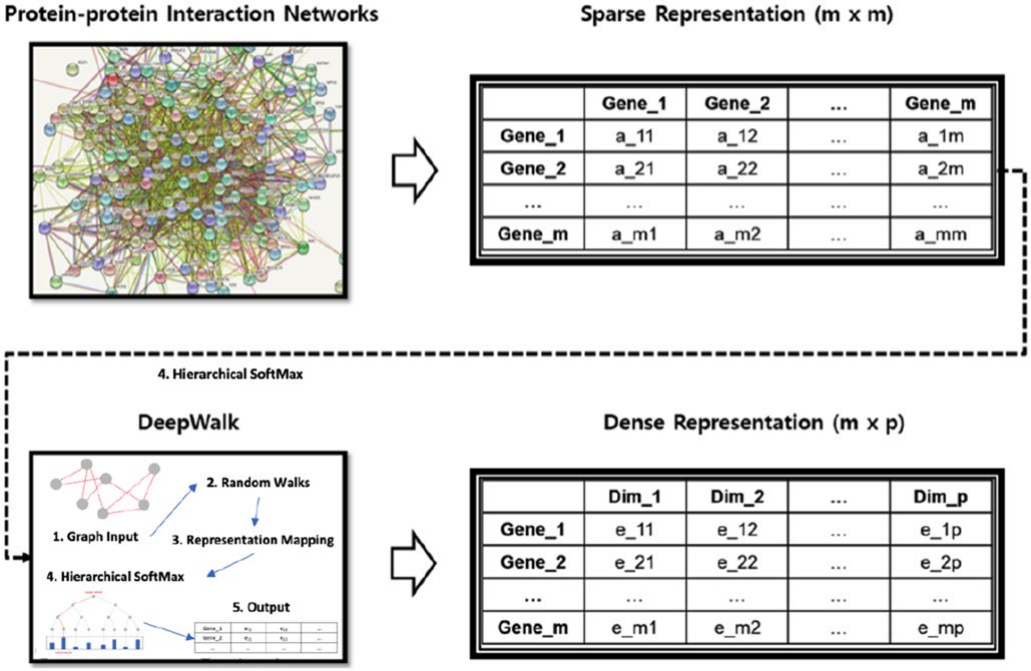
Extracting subnetworks using graph embedding technique . This involves 1) generating an adjacency matrix from a given PPI network, 2) random work sampling from a given PPI graph, and 3) generating a word2vec representation of the sampled works to generate a dense representation of each gene.

Extracting subnetworks from a given PPI network , taking into account its biological significance, is an important task in constructing subnetwork-level representations. Essentially, the problem can be thought of as a clustering node within a PPI network represented in the form of an adjacency matrix (Fig. 1). The sparsity of network representation is useful for defining clusters, but at the same time is a huge challenge to the generalization of machine learning. DeepWalk is a powerful tool to deal with this problem, deploying representation learning techniques based on neural networks such as Word2Vec^18, 22^. It works as a graph embedding tool and shows good performance when used for node classification^18^. In the study of Perozzi et al.^18^, DeepWalk was compared with five other methods in terms of the multi-label classification task, which is a problem of erasing some of the labels and guessing the erased labels through node clustering when given a graph with labels on each node. DeepWalk outperformed all other opponents under various experimental conditions. Based on the Macro-f1 score, DeepWalk’s performance reaches up to 43.05%.

**Figure 2:**
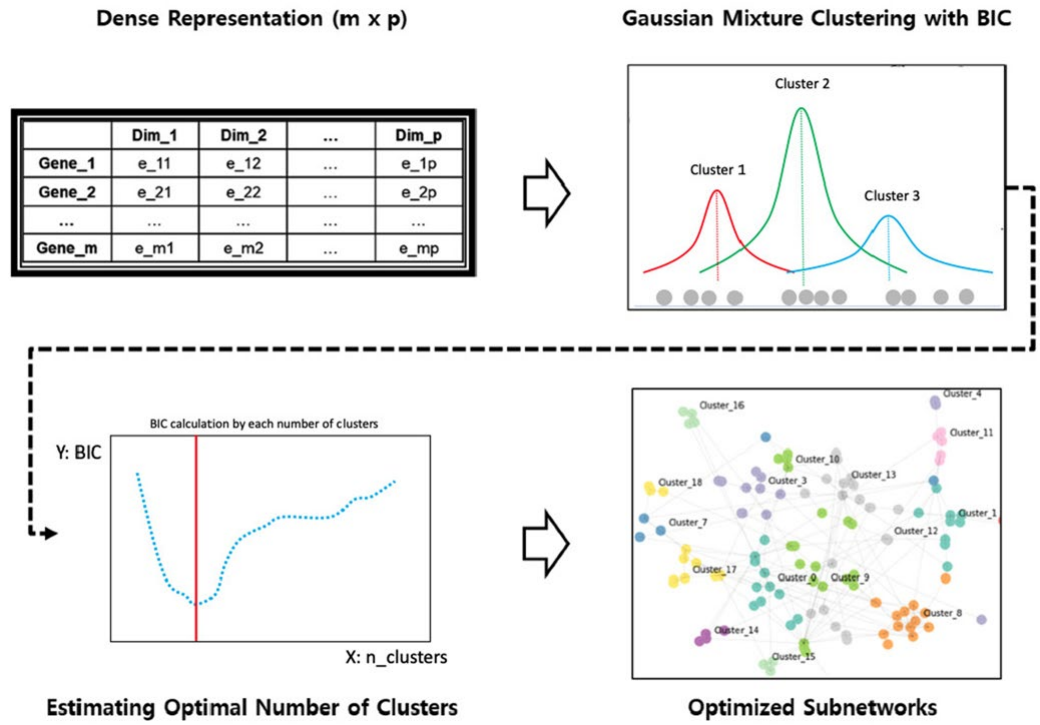
Subnetwork clustering using latent representation. This involves (1) applying a Gaussian mixture model to a given dense representation of the PPI network using a wide range of the number of components as parameters, (2) evaluating each model by calculating the BIC criterion, and (3) choosing the best model to create a subnetwork for a given PPI network.

DeepWalk receives the sparse representation of the PPI network and generates a dense representation of the individual nodes encoding the relationship between each node in a continuous vector space with a reduced number of dimensions (Fig. 1). Using the encoded vector as a new representation of each node, we can solve the problem of extracting subnetworks by transforming it into a clustering problem. We used the Gaussian Mixture Model (GMM) and Bayesian Information Criteria (BIC) (Eq. 1) to estimate the optimal number of clusters for a given PPI network , and each resulting cluster can be considered a subnetwork (Fig. 2). For this step, the Python library scikit-learn-0.19.2 was used^23^.

1 $$\begin{aligned} BIC =\ln {(n)}{(kd)}-2\ln {(p(x\mid \widehat{\theta },M))} \end{aligned}$$

where *x* is the observed data, *n* is the number of data points in *x*, and *k* is the number of clusters. *d* is the number of dimensions of the latent representation generated by DeepWalk. $p(x\mid \widehat{\theta }, M)$ represents the maximum value of the GMM likelihood function. Where $\widehat{\theta }$ is the parameter value that maximizes the likelihood function. The model with the lowest *BIC* value is considered optimal.

**Figure 3:**
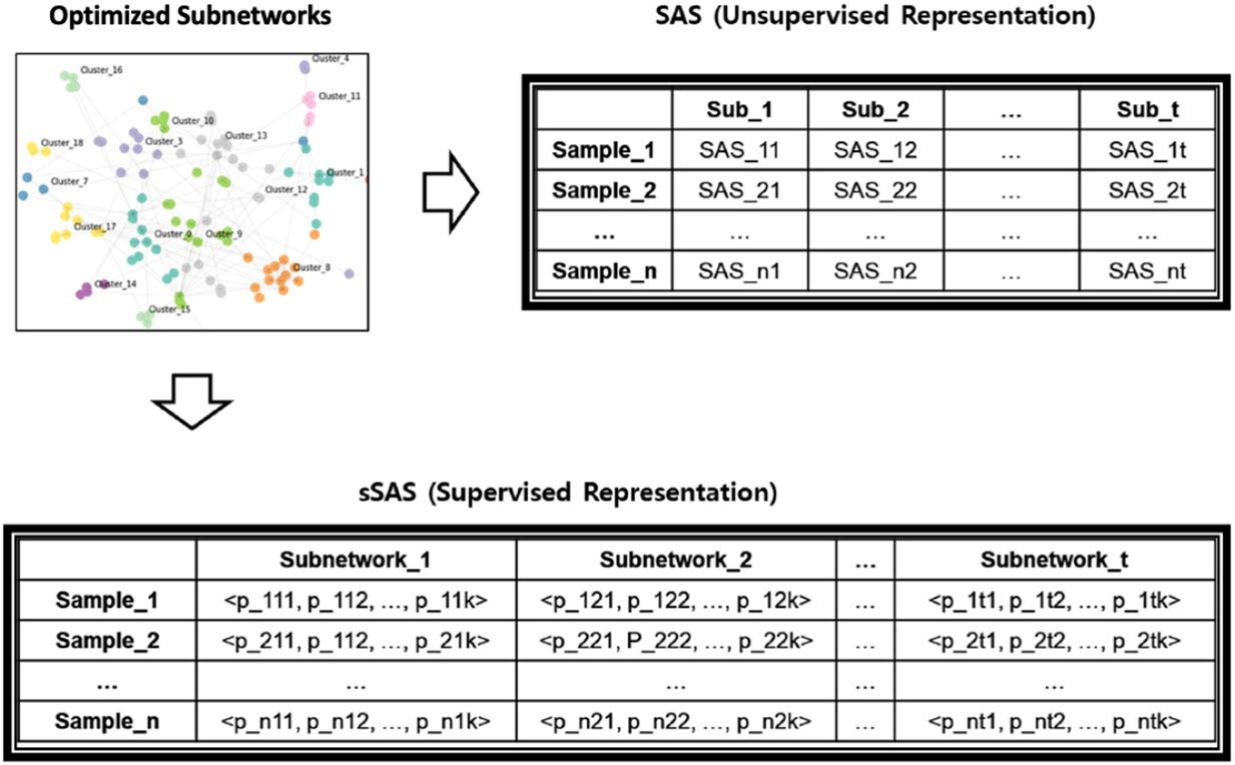
Constructing subnetwork level representation. This includes (1) calculating the sSAS representation for each optimized subnetwork and (2) integrating the representations into the subnetwork-level representation for each sample.

In order to improve interpretability and reduce noise, we took the Hallmark Gene Set (HGS) from the molecular signature database (MSigDB)^24^ to limit the gene space. HGS is a well-selected group of functional genes, in which genes associated with a common cancer phenotype are grouped into a set of genes. HGS has 50 genesets containing a total of 4,384 genes. For each geneset, we first generated a PPI network graph using the protein-protein interactions between the genes of each geneset. Here , PPI networks were extracted from BioGrid^21^ using only high-confidence protein interactions . For each PPI network graph, we applied DeepWalk to create a vector space, then applied GMM to create subnetworks (Figs. 1 and 2). By defining sub-networks within each HGS geneset, the genes in each sub-network are not only closely linked in terms of PPI network , but also in terms of cancer phenotype. In summary, 279 subnetworks were obtained, each subnetwork assigned to one of the 50 HGS genesets.

### Construction of subnetwork level representation

Constructing a subnetwork-level representation of cancer transcriptome requires the integration of gene expression levels and PPI networks between genes so that the activity of each subnetwork can be quantified. SAS is one of the most effective tools to this end^19^. SAS uses RNA-seq samples and subnetworks generated from PPI networks as inputs to quantify subnetwork level activation for each sample. As explained in the Eqs. (2a), (2b), (2c) and (2d), SAS is a single value called Subnetwork Activation Score for the subnetwork level representation of the transcriptome. It is defined as a nonlinear combination of gene expression using the closeness centrality of each gene with a coefficient defined by a given PPI network .

2a $$\begin{aligned}&ACT_{i,j} = N_{i,j} * \frac{{(c_{i}r_{i}+c_{j}r_{j})}^2}{2(r_{i}+r_{j})} \end{aligned}$$

2b $$\begin{aligned}&SAS = \sum _{i}\sum _{j}ACT_{i,j} \end{aligned}$$

2c $$\begin{aligned}&N_{i,j} = \frac{a_{ij}}{\sum _{s}\sum _{t}a_{st}} \end{aligned}$$

2d $$\begin{aligned}&a_{ij} = {\left\{ \begin{array}{ll} 1 &{} \text {if gene i and j are connected} \\ 0 &{} \text {otherwise} \end{array}\right. } \end{aligned}$$

$ACT_{i,j}$ represents the edge level activation score between the two genes *i* and *j*. $r_{i}$ represents the expression level of the gene *i* (TPM in this case). $c_{i}$ represents the closeness centrality of the gene *i* within a given subnetwork PPI network . *SAS* is the total activation score for the subnetwork. $a_{i,j}$ is an indicator of whether two genes are linked within a given PPI network , and $N_{i,j}$ is the normalized term for $a_{i,j}$.

While SAS does not use sample labels when calibrating subnetwork representations, our goal is to predict metastasis potential in early OTSCC, so we modified SAS to better serve this purpose and named it supervised SAS (sSAS). sSAS inherited the basic idea of SAS, but calculated the coefficients in different ways (e.g. $c_{i}$ and $N_{i,j}$). Rather than defining the coefficients directly in the network topology, they were estimated by maximizing the log-likelihood function (Eq. 3f) designed to be considered as a latent variable and minimize prediction errors for the labels of each sample. As shown in Eq. (3a) and (3b), *sSAS* is defined as a logit in a logistic regression problem rather than a single activation score. *x* is defined as a vector containing a nonlinear combination of gene expression combined by paired combinations of genes, and $\theta$ is the latent weight corresponding to *x*. The problem definition is as follows.

First, the $ACT_{i,j}$ term is divided into three parts: $\frac{r_{i}^2}{r_{i}+r_{j}}$, $\frac{r_{j}^2}{r_{i}+r_{j}}$, and $\frac{r_{i}r_{j}}{r_{i}+r_{j}}$. Then, all coefficients are considered as latent variables such as $w_{ij1}$, $w_{ij2}$, and $w_{ij3}$. Then the linear combination of the three division terms replaces $ACT_{i,j}$ (Eq. 3a). We named it $sACT_{i,j}$ as a supervised $ACT_{i,j}$. Then the term *SAS* is also changed to a supervised format (e.g. *sSAS*) to estimate the latent weights by target variable (ie, sample label) (Eq. 3b). The original observations are transformed into a nonlinear combinatorial vector of gene expression *x* (Eq. 3c) and their weights are defined by the model parameter $\theta$ (Eq. 3d). Based on this, the logistic function $q_{k}(x)$ is defined to represent the estimated probability of observation *x* with target label *k* (Eq. 3e). Finally, a log-likelihood function $l(\theta _{k})$ is defined so that the model parameter $\theta _{k}$ can be estimated by maximizing $l(\theta _{k})$ (Eq. 3f and 3g).

3a $$\begin{aligned}&sACT_{i,j} = w_{i j 1}(\frac{r_{i}^2}{r_{i}+r_{j}}) + w_{i j 2}(\frac{r_{j}^2}{r_{i}+r_{j}}) + w_{i j 3}(\frac{r_{i}r_{j}}{r_{i}+r_{j}}) \end{aligned}$$

3b $$\begin{aligned}{}&sSAS = \ln {\frac{q}{1-q}}= \sum _{i}\sum _{j}sACT_{i,j} \end{aligned}$$

3c $$\begin{aligned}&x = \left\langle \frac{r_{1}^2}{r_{1}+r_{2}}, \frac{r_{2}^2}{r_{1}+r_{2}}, \frac{r_{1}r_{2}}{r_{1}+r_{2}}, ... \right\rangle \end{aligned}$$

3d $$\begin{aligned}&\theta = \langle w_{ij1}, w_{ij2}, w_{ij3}, ... \rangle \end{aligned}$$

3e $$\begin{aligned}&q_{k}(x) = \frac{1}{1+e^{-{\theta _{k}^{T}x}}} \end{aligned}$$

3f $$\begin{aligned}{}&l(\theta _{k}) = \sum _{m}y_{mk}\ln {q_{k}(x_{m})} + (1-y_{mk})\ln (1-q_{k}(x_{m})) \end{aligned}$$

3g $$\begin{aligned}&y_{mk} = {\left\{ \begin{array}{ll} 1 &{} \text {if the label of sample m is k} \\ 0 &{} \text {otherwise} \end{array}\right. } \end{aligned}$$

The representation of a subnetwork *t* of a sample *m* is defined in Eq. (4a) and (4b). In the case of multiple classes, the model parameter for each class $\theta _{k}$ is independently estimated in a “one versus the rest” way, then consolidated into $p_{mtk}$ as in Eq. (4b). In our scheme, therefore, the subnetwork level representation of a sample is probability distribution estimated from the given data at each subnetwork by logistic regression model (Eq. 4c and Fig. 3). For example, if RNA-seq samples have *k* classes of labels then each RNA-seq sample will have a vector with dimensions of $279 * k$ because we used 279 subnetworks in this study. scikit-learn-0.19.2 was used for this step^23^.

4a $$\begin{aligned}&q_{mtk} = \frac{1}{1+e^{-\theta _{k}^{T}x_{m}}} \end{aligned}$$

4b $$\begin{aligned}&p_{mtk} = \frac{q_{mtk}}{\sum _{r}{q_{mtr}}} \end{aligned}$$

4c $$\begin{aligned}&Sub_{mt} = \langle p_{mt1}, p_{mt2}, ..., p_{mtk} \rangle \end{aligned}$$

### Integration of subnetwork level representation into master-level decision

The remaining problem is to incorporate the constructed subnetwork level representation into a single master level decision. We solved this ensemble learning problem using the attention layer built into the neural network. The attention mechanism stems from the problem of sequence-to-sequence mapping in machine translation^25^. In the work of Bahdanau et al., the attention layer was inserted between the encoder and decoder layer to act as memory^25^. In other words, they are trained to dictate which context to focus on at a specific point in time and which context to not. The attention mechanism has been applied to various tasks and has been shown to exhibit excellent performance^26, 27^. Also, Choi et al.^27^ suggested that the attention mechanism can be used to make models more explainable. In this model, the attention layer acts as a master-level decision agent trained to decide which sub-network to focus on based on the certainty computed with each sub-network level representation (Fig. S1). As shown in Eq. (5a) and (5b), the attention layer takes the negative Shannon’s entropy^28^ of each subnetwork level representation. Since the entropy of a given probability distribution represents the level of uncertainty, negative entropy was used to quantify how certain each subnetwork level predictor is for a given classification task.

The negative entropy values of each subnetwork are concatenated into a single vector (Eq. 5b) and passed to the next fully concatenated layer (FC). Then softmax activation is applied, resulting in a proportional distribution that is the attention layer (Eq. 6a and 6b). Hence, the actual parameter that can be learned here is the *W* matrix (Eq. 6a), which learns to decide which subnetworks to focus on based on the *C* vector. The actual decision-making process is described in Eq. (6c) and (6d) (Fig. S2). It is basically the weighted sum of subnetwork level representation for each class, where the weights are learned by the attention mechanism. The prioritization of features by the model is an instance-wise process, so each sample gets a different attention value depending on their subnetwork level representation. Python libraries tensorflow-1.10.0^29^ and keras-2.2.2^30^ were used for this step.

5a $$\begin{aligned}&c_{t} = \sum _{k}{p_{tk}\ln {p_{tk}}} \end{aligned}$$

5b $$\begin{aligned}&C = \langle c_{1}, c_{2}, ..., c_{t} \rangle \end{aligned}$$

6a $$\begin{aligned}&H = softmax(WC^{T}) \end{aligned}$$

6b $$\begin{aligned}&H = \langle h_{1}, h_{2}, ..., h_{t} \rangle \end{aligned}$$

6c $$\begin{aligned}&d_{k} = \sum _{t}{h_{t}*p_{tk}} \end{aligned}$$

6d $$\begin{aligned}&f_{k} = \frac{e^{d_{k}}}{\sum _{s}{e^{d_{s}}}} \end{aligned}$$

## Evaluation design

Two sets of experiments were prepared to evaluate the proposed method. 1) The first used breast invasive carcinoma (BRCA) cohort data from the Cancer Genome Atlas (TCGA) consortium^31^ (referred to as BRCA-case). 2) The second used squamous cell carcinoma of the head and neck (HNSC) cohort data from TCGA^32^ and proprietary data provided by SMG-SNU Boramae Medical Center (referred to as ORAL-case). The purpose of the first experiment was to test the model’s performance with a well-known dataset so that the results could be compared to previously studied knowledge that corresponds to the case. The second experiment was the main subject of the study.

### Data description: BRCA-case

For BRCA-case, we collected 981 RNA-seq samples from TCGA, where each of them is labeled with PAM50 classes^33^. PAM50 is a standard *de facto* method for identifying the molecular status of breast cancer, which has five subtypes: lumen A (LumA), lumen B (LumB), HER2-enriched (HER2), basal (Basal), and normal (Normal) subtypes, which was initially defined by unsupervised clustering analysis using a whole-genome scale gene expression profile. There were 499 LumA, 197 LumB, 78 HER2, 171 Basal, and 36 Normal . Note that they were all primary tumors and samples with the Normal subtype were excluded. Note that all the RNA-seq samples have gene expression levels measured by the Transcripts Per Million (TPM) scale for 20,501 genes.

### Data description: ORAL-case

For ORAL-case, we collected 97 RNA-seq samples from both TCGA and SMG-SNU Boramae Medical Center, where 64 RNA-seq samples (will be referred to as TCGA-ORAL samples) were from TCGA and 33 RNA-seq samples (will be referred to as SNUH-ORAL samples) were from SMG-SNU Borame Medical Center. Each of them was primary tumors with oral tongue origin and cancer stages I and II. They were labeled with their lymph node metastatic status as Positive and Negative. For TCGA-ORAL samples, there were 31 samples labeled with Positive and 33 samples labeled with Negative, while there were 11 samples labeled with Positive and 22 samples labeled with Negative in SNUH-ORAL samples. The gene expression levels were measured the same as in the BRCA-case.

### Validation

For each experiment, we divided the dataset into train and test sets to validate the performance of the method. In BRCA-case, we randomly sampled 30% of the overall samples with class label balanced and considered them as a test set. In summary, 689 samples (LumA: 350 , LumB: 138 , Her2: 55, Basal: 120, and Normal: 26 ) were used as a train set, while 292 samples (LumA: 149 , LumB: 59 , Her2: 23, Basal: 51, and Normal: 10 ) were used as a test set. In ORAL-case, the 64 TCGA-ORAL samples (Positive: 31 and Negative: 33) were used as a train set, while the 33 SNUH samples (Negative: 22, Positive: 11) were used as a test set. Note that the SNUH samples had completely different geographical and ethnic compositions (ie, Korean) compared to the TCGA samples in ORAL-case. Three types of metrics were used in the evaluation. 1) Averaged area under the curve (mAUC) , 2) Accuracy (ACC), and 3) F1 score (F1). mAUC is the adjusted value of AUC for class imbalance in a multiclass classification problem. mAUC is the average AUC for each class when treated as a binary classification (i.e. one versus the rest). The mAUC, ACC, and F1 measured with the training set are $mAUC_{train}$ , $ACC_{train}$, and $F1_{train}$ and the values measured with the test set are $mAUC_{test}$ , $ACC_{test}$, and $F1_test$ .

### Prediction power evaluation

For evaluation, we first set the baseline performance using existing machine learning methods such as Logistic Regression (LR), K Nearest Neighbors (KNN), Random Forest (RF), Support Vector Machine (SVM) , and Multi-Layer Perceptron (MLP) in each case (ie, BRCA-case and ORAL-case). The experimental setup was prepared with all possible combinations of parameters listed in Table S2. The total number of combinations was 25,664. We chose the model with the best performance in terms of mAUC and mACC and used it as the baseline performance to evaluate the proposed method. scikit-learn-0.19.2 was used for this test^23^. Similarly, we evaluated all the possible combinations of the parameters listed in Table S1 to select the parameters to be used in the proposed method. The total number of combinations was 4,900. By comparing the best performances between the conventional methods and our method after searching for each parameter space with comparable sizes, we can estimate the extent to which our method can perform relatively better than conventional methods. The python library tensorflow-1.10.0^29^ and keras-2.2.2^30^ were used for this setup.

### Network biomarker evaluation

The attention layer assigns weights to each subnetwork level prediction (Eqs. 6a, 6b and 6c), where the weights are the probability distribution due to softmax activation. Therefore, each weight can be considered the importance of the features learned in the decision model (Fig. S2). The weights are defined instance-wise, so the overall feature importance (i.e. $FI_t$) was defined as the average value of all samples (Eq.7). Then, each subnetwork was ranked in ascending order. The feature ranking of the subnetwork *t* is called $FIR_t$.

7 $$\begin{aligned} FI_t = \frac{\sum _{n=1}^{N}{h_{nt}}}{N} \end{aligned}$$

Note that the $h_{nt}$ indicates the attention value of the subnetwork *t* of sample *n*.

We conducted a test to evaluate how well the decision model prioritizes features. In BRCA-case, since the PAM50 subtyping is based on the 50 genes^33^, we can use this information to design a permutation test. First, we defined a function that defines a score for the reference geneset (ie, PAM50 genes) against a given feature rankings of subnetworks (Eqs. 8a, 8b, and 8c). Then, the score of PAM50 genes with the feature rankings provided by the decision model was set as a baseline score (Eq. 8a). Next, a permutation test was performed with feature rankings shuffled 1-million times (Eqs. 8d, 8e, 8f, and 8g). The purpose of shuffling is to simulate the null hypothesis by generating randomized rankings. Then, the number of randomized settings that exceed the baseline score was counted for calculating empirical p-value (Eq. 8d). The purpose of this test was to evaluate how significantly the feature rankings learned by the attention layer reproduce the prior knowledge corresponding to the given data (ie, PAM50 genes).

8a $$\begin{aligned}&SCORE = \frac{\sum _{g=1}^{50}{RANK_{g}}}{50} \end{aligned}$$

8b $$\begin{aligned}&RANK_{g} = \frac{\sum _{t}{SUB_{gt}*FIR_{t}}}{\sum _{t}{SUB_{gt}}} \end{aligned}$$

8c $$\begin{aligned}&SUB_{gt} = {\left\{ \begin{array}{ll} 1 &{} \text {if gene g is included in subnetwork t} \\ 0 &{} \text {otherwise} \end{array}\right. } \end{aligned}$$

8d $$\begin{aligned}&p\text {-}value = \frac{\sum _{iter=1}^{1,000,000}{I_{iter}}}{1,000,000} \end{aligned}$$

8e $$\begin{aligned}&I_{iter} = {\left\{ \begin{array}{ll} 1 &{} \text { if } SCORE_{iter}^{permute} > SCORE \\ 0 &{} \text {otherwise} \end{array}\right. } \end{aligned}$$

8f $$\begin{aligned}&SCORE_{iter}^{permute} = \frac{\sum _{g=1}^{50}{RANK_{g,iter}^{permute}}}{50} \end{aligned}$$

8g $$\begin{aligned}&RANK_{g,iter}^{permute} = \frac{\sum _{t}{SUB_{gt}*FIR_{t,iter}^{permute}}}{\sum _{t}{SUB_{gt}}} \end{aligned}$$

## Results

### BRCA-case

**Figure 4:**
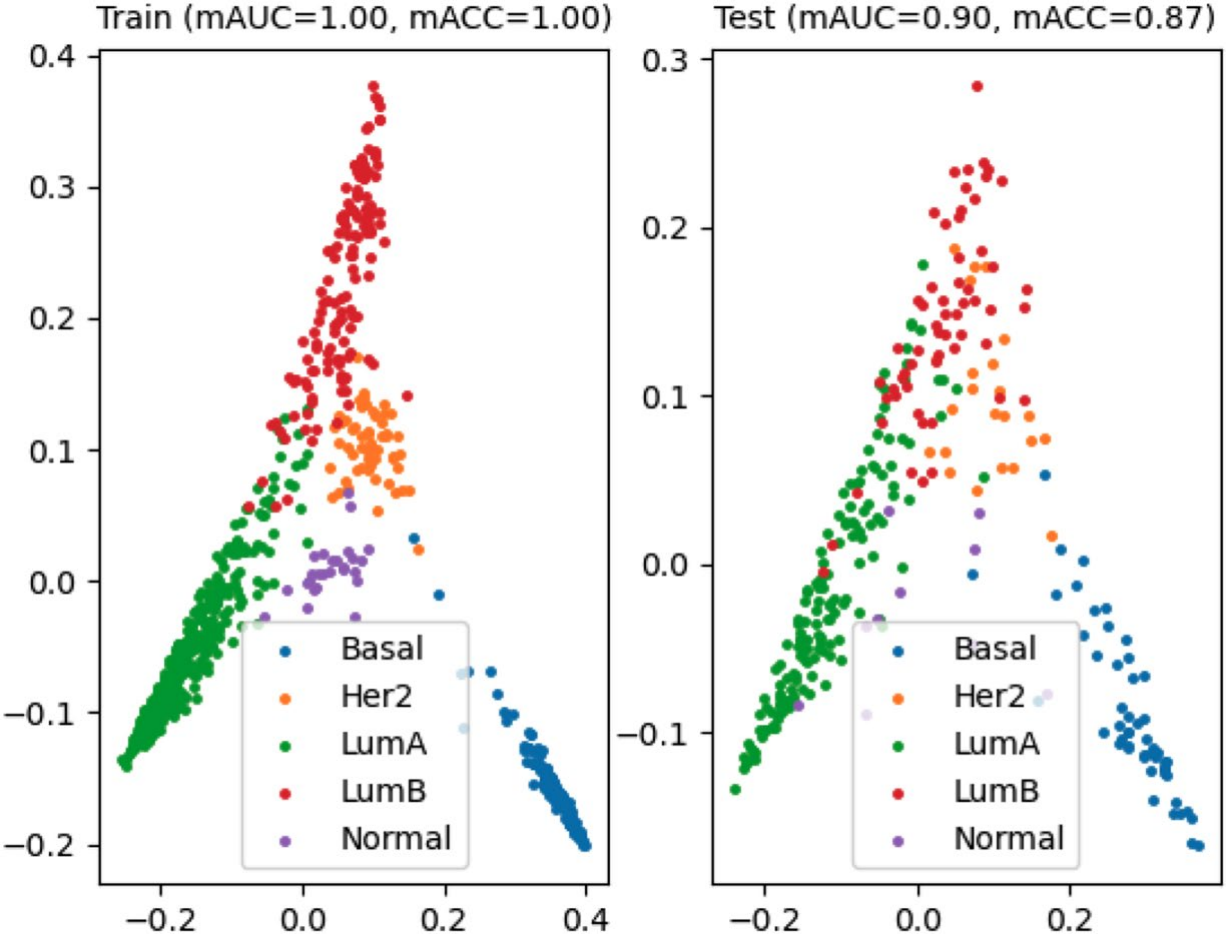
Performance evaluation results for PAM50 subtype prediction in breast cancer. The color-coding indicates the actual class label of samples.

As mentioned, we designed an evaluation scheme for comparing the baseline methods and the proposed method in terms of mAUC and mACC. As noted, the figures below are the results of PAM50 label predictions for the TCGA-BRCA cohort. In BRCA-case, the SVM algorithm with cosine kernel PCA with 18 components, TPM legalization, and no feature selection showed the best performance ($mAUC_{test}$: 0.8700, $mACC_{test}$: 0.8390, and $F1_{test}$: 0.8320 ) for the baseline performance. Our method showed a better performance ($mAUC_{test}$: 0.9006, $mACC_{test}$: 0.8664, and $F1_{test}$: 0.8623 ) (Fig. 4), using adagrad, squared hinge loss, 10% dropout, 25% split, and with feature selection. Also, we conducted the permutation test over the feature rankings that were generated by the attention layer of the best model, which showed that PAM50 genes are significantly highly ranked in the resulted attention layer (empirical p-value: 1.1e−05). See the resulting confusion matrix of our approach in Supplementary Table S3.

### ORAL-case

**Figure 5:**
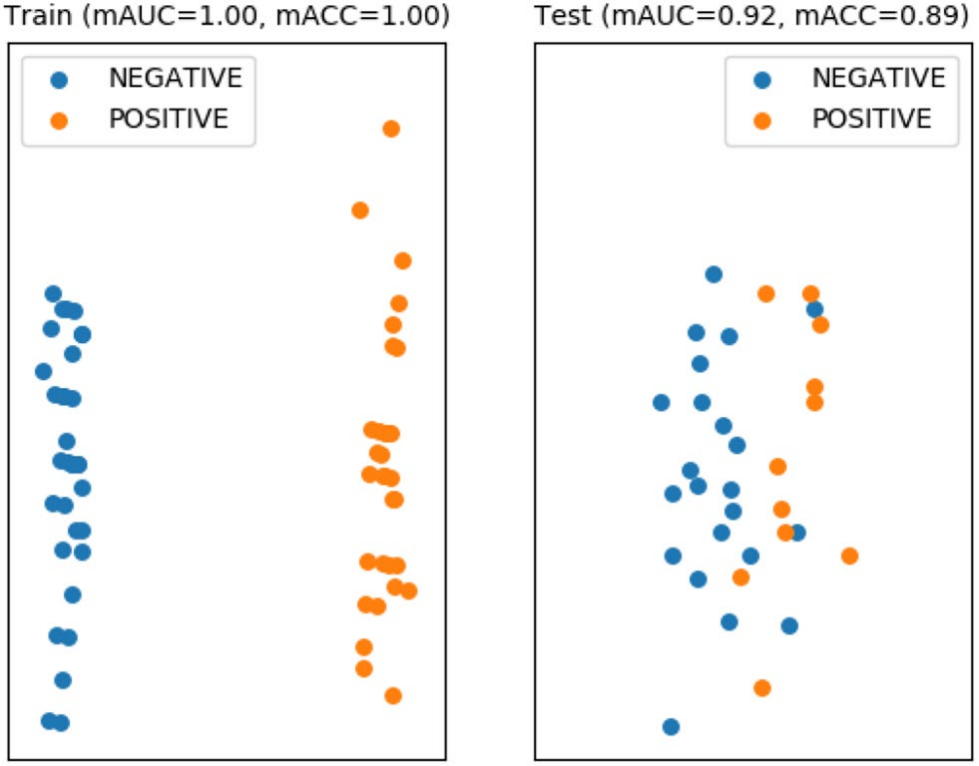
Performance evaluation results for lymph node metastasis prediction in early oral tongue cancer. The color-coding indicates the actual class label of samples.

In ORAL-case, the RF algorithm with linear kernel PCA, dimension reduction size 4, TPM logarization, and using feature selection showed the best performance ($mAUC_{test}$: 0.7045, $mACC_{test}$: 0.7576, and $F1_{test}$: 0.7570 ) for the baseline performance. This is the result of predicting lymph node metastasis in OTSCC. Our method showed better performance ($mAUC_{test}$: 0.9174 , $mACC_{test}$: 0.8864 , and *F*1: 0.8333 ) (Fig. 5), using AdaGrad, Mean absolute percentage error, 50% dropout, 10% split, and a feature selection. The more detailed metrics are in Table 1 and the top-5 subnetworks highlighted by the attention layer are in Table 2. See the resulting confusion matrix of our approach in Supplementary Table S3.

**Table 1:** Detailed metrics for lymph node metastasis prediction in early oral tongue cancer.

Measure	Value*	Description
*TP*	10	# of True positive
*TN*	19	# of True negative
*FP*	3	# of False positive
*FN*	1	# of False negative
Sensitivity	0.9091	$TPR = \frac{TP}{TP + FN}$
Specificity	0.8636	$SPC = \frac{TN}{FP + TN}$
Precision	0.7692	$PPV = \frac{TP}{TP + FP}$
Negative predictive value	0.9500	$NPV = \frac{TN}{TN + FN}$
False positive rate	0.1364	$FPR = \frac{FP}{FP + TN}$
False discovery rate	0.2308	$FDR = \frac{FP}{FP + TP}$
False negative rate	0.0909	$FNR = \frac{FN}{FN + TP}$
Accuracy	0.8788	$ACC = \frac{TP + TN}{P + N}$
F1 score	0.8333	$F1 = \frac{2TP}{2TP + FP + FN}$

* Note that the metrics are not class-balanced.

**Table 2:** Attention map of the best model using the proposed method.

Subnetwork^1^	Attention^2^ (%)	Genes
EPITHELIAL_MESENCHYMAL		
_TRANSITION_2	9.12	BMP1,DAB2,FBLN5,GADD45A,GEM,LOXL1,LUM,SNAI2,TPM1, VEGFA
E2F_TARGETS_5	9.06	AURKB,BRCA1,CCNE1,CDC20,CDKN2C,EXOSC8,GINS3,IPO7, MAD2L1,MCM7,POLA2,PRIM2,PTTG1,RAD1,RAD21,RANBP1, SYNCRIP,TK1,TUBB,XPO1
MYOGENESIS_6	7.97	ADAM12,CDKN1A,HSPB8,KIFC3,MB,MYOZ1,PSEN2,TNNC2, TNNT3,TPM3
TNFA_SIGNALING_VIA_NFKB_6	7.45	CCND1,CEBPB,CFLAR,ETS2,FOS,GADD45A,MAP3K8,MYC, NFE2L2,SMAD3,SPHK1,TNF,TRAF1,TRIB1
MITOTIC_SPINDLE_2	4.78	ARHGAP27,ARHGEF11,CENPE,CEP250,KIF4A,KIF5B,KIFAP3, LLGL1,LMNB1,RACGAP1,RASA1,TUBA4A

Note that the top-5 subnetworks are listed.

^1^SUBNETWORK column indicates the name of subnetworks, where the prefix is the name of HGS geneset and the postfix (the integer number) is the index.

^2^ATTENTION column indicates the attention values generated by the best model of the proposed method averaged by all samples (including the TCGA and SNUH samples).

## Discussion

As described, the proposed method outperformed the baseline methods in both BRCA-case and ORAL-case, and the attention map of the best model was found to greatly reproduce the prior knowledge in BRCA-case. This indicates that our method can construct a computational model for predicting patient status based on the subnetwork level representation of transcriptome. Also, the proposed method outperformed the baseline method in ORAL-case, showing that the model constructed by our method is robust enough to be reproduced between two groups of different geographical and ethnic origins.

### Robustness of the method

Since there were not many samples available (64 for ORAL), two additional evaluations were performed instead of cross-validation to determine if the method was overfitting. In BRCA-case, we performed a permutation test to determine how the PAM50 gene was enriched in the resulting attention map of the best model, as described in the “Network biomarker evaluation” section. The PAM50 subtype is based on 50 genes, so if the model truly reflects the underlying biology and is not overfitted to noise, those 50 genes should rank higher than the others. In this regard, the results in the“ BRCA-case” section clearly show that the model did not overfit in the BRCA-case (permutation test p-value: 1.1e−05).

In ORAL-case, we performed an extensive literature search to find out how the high-rank sub-networks in ORAL-case are associated with lymph node metastasis in OTSCC. The literature search results strongly suggest that the top-ranked subnetworks are closely linked with lymph node metastasis. This also implies that the model is not overfitting. The attention layer were able to capture core mechanisms known to be associated with lymph node metastasis in OTSCC as well as other cancer types, which are listed as follows.

1. Epithelial-Mesenchymal Transition (EMT, 9.12%): EMT is a series of critical events observed during cancer progression including invasion and metastasis caused by the acquisition of fibroblast-like phenotype of cancer cells, which is the core mechanism of lymph node metastasis in various types of carcinoma including OTSCC^34^.
2. E2F Targets (9.06%): E2F is a class of transcription factors that regulates the expression of genes associated with cell proliferation^35^, which has been known to affect the Disease-Free Survival (DFS) in oral cancer^36^.
3. TNF$\alpha$ Signaling via NF$\kappa$B (7.45%): Tumour Necrosis Factor Alpha (TNF$\alpha$) is an import inflammatory factor that has a critical role in proliferation, migration, invasion, and angiogenesis, which frequently collaborates with the Nuclear Factor Kappa B (NF$\kappa$B), inducing tumor cell invasion and metastasis^37^. The TNF$\alpha$ and NF$\kappa$B signaling have been known to be associated with invasion and metastasis in oral cancer^38^.

The highlighted subnetwork of the attention map showed a strong association with the results of previous studies, suggesting that the model is very reliable in terms of consistency with prior knowledge. This also suggests that other subnetworks (eg, Mitotic spindle and Myogenesis) may be unknown regulators of lymph node metastasis in OTSCC. A complete list of the attention maps for BRCA-cases and ORAL-cases is listed in Table S3.

### Biological significance of the subnetworks of PPI network extracted by DeepWalk

In our method, a neural network-based graph embedding technique DeepWalk^18^ was used for extracting subnetworks from a given PPI network . DeepWalk generates vectors of real numbers for each protein in a given PPI network , where vector distances between adjacent proteins in the PPI network are smaller than the distant proteins. Hence, clusters generated by using the DeepWalk representation can be considered as well-optimized collections of interacting proteins in terms of the PPI network . It has been known to show better performances than the classical graph clustering approaches such as SpectralClsutering and Modularity-based clustering^18^. The strength of DeepWalk comes from the random-walk-based estimation of topological distances between two proteins, meaning that it considers not only the direct edges between two proteins but also considers indirect connections implied in the neighborhood information between two proteins. It allows the model to capture the hidden relationships between two proteins, which might not be explicitly specified in the PPI network due to the lack of knowledge.

In addition, we used MSigDB Hallmark Gene Set (HGS) to annotate sub-networks, a set of cancer hallmark genes, so the framework is currently only valid for cancer tissues. It is designed to work for any cancer tissues, as it has been shown to be effective in both oral and breast cancers.

### Advantages of the supervised setting for constructing subnetwork level representations

Originally, the SAS^19^ method was not designed for a classification problem. Hence, we replaced the coefficients in the SAS framework with latent variables estimated by using each sample label (Eq. 3f). It virtually rewires edges within each subnetwork to be suited for solving the given classification problem. There are three advantages to this setting. First, it can fill the gap of knowledge from the data, such as unknown interactions between proteins. Secondly, it can calibrate the edge weights to reflect condition-specific interactions or broken interactions that are specific to given data. Lastly, it can reduce the weights of passenger interactions, redistributing to the drivers^39^.

There are many dimensionality reduction techniques such as principal component analysis (PCA)^40^ . The main difference between PCA and SLR is the interpretability of the results each model produces. For PCA, the output is just a coefficient assigned to each gene to transform each sample’s gene expression vector into a reduced vector embedding space. It is difficult to infer biological knowledge or therapeutic targets from the results. On the other hand, our approach can generate subnetwork-level attention maps highlighting the subnetworks that are important for predicting specific cancer phenotypes. It is much more intuitive and informative in terms of biological and clinical applications.

### Clinical implication of the decision making process by the attention layer

In our method, the final prediction was made by combining each subnetwork level representation, which can be considered a type of multimodal learning. The multimodal learning approach has been applied to cancer genomics as a tool to integrate heterogeneous data sources such as multi-omics integration^41^. In our model, subnetworks that are optimally defined in terms of PPI network and HGS are considered multimodal units, meaning that each subnetwork level prediction has been independently generated to recognize patient status. This is not a new concept on the clinical bench. Clinicians routinely use heterogeneous sources of information to make well-adjusted decisions^42^. Thus, the human clinician’s decision-making process is inherently multimodal. Our model can be considered a computationally well-optimized tool for simulating the decision-making process of human clinicians based on transcriptome data. In addition, explainability is a key challenge for maximizing the utility of a machine learning model^43^. Reducing high dimension transcriptome data into a much smaller but biologically meaningful subnetwork space will be helpful to explain the prediction result in a more acceptable way for both clinicians and patients. Moreover, since the attention layer operates in an instance-wise manner, the attention map of each patient represents the highlights of the importance of each subnetwork customized to each patient, which can be useful for the personalized medicine^44^. Lastly, even though the attention map mostly focused on the subnetworks well-known to be associated with metastasis, some of them (eg, Mitotic spindle and Myogenesis) were relatively not studied for their connection to the metastasis. It suggests that the attention model can be used as a tool for discovering previously unknown network biomarkers, which can lead to a new understanding of cancer biology or new therapeutic targets.

### Limitations and future works

As stated in “Biological significance of the subnetworks of PPI network extracted by DeepWalk”, the HGS geneset is pre-customized for each cancer phenotype, so the proposed method is essentially tailored only to cancer samples. Although it is designed to act on all cancer tissues, it has only been tested for oral and breast cancers, so its applicability to other cancer types is unknown. To clarify this, future experiments need to be conducted to apply the proposed method to a wide range of cancer samples, such as the pan-cancer project $^?$ data.

## Conclusion

The proposed method is a computational framework that learns subnetwork representations of the transcriptome to discover network biomarkers and determine metastatic potential in early oral tongue squamous cell carcinoma. This method achieved high accuracy in predicting the likelihood of metastasis in two geographically and racially different groups of patients. The robustness of the model and the reproducibility of the discovered network biomarkers show great potential as a tool to diagnose lymph node metastasis in early oral cancer. Our contribution can be summarized as follows .

1. Developed a method to define optimized subnetworks from a given PPI network using a state-of-the-art graph embedding technique.
2. Developed a supervised subnetwork representation learning system to successfully reduce the input dimension of transcriptome data by considering complex dependencies between genes, leading to robust prediction models with good performances.
3. Developed an attention-based classification system to integrate the subnetwork level representations, creating an attention map that highlights important network biomarkers tailored to each patient, leading to feature rankings that significantly reproduced the prior knowledge.

## Supplementary Information

Supplementary Information 1.

Supplementary Information 2.

## Data Availability

TPM-quantified gene expression levels for TCGA-BRCA and TCGA-HNSC were downloaded from http://firebrowse.org/, where the archive name is illuminahiseq_rnaseqv2-RSEM_genes in the mRNASeq section. The PAM50 label of TCGA-BRCA was extracted from https://www.cbioportal.org/study/summary?id=brca_tcga_pan_can_atlas_2018. The OTSCC annotation information such as tissue origin and the tumor stage is based on the clinical information of the TCGA-HNSC cohort downloaded from http://firebrowse.org/, where the archive name is Clinical_Pick_Tier1 in the Clinical section. A cleaned dataset is deposited on Zenodo, whose link is “https://zenodo.org/record/5485336#.YTfuPp5Kiqk”. All the other related data and source codes are available at https://github.com/mdy89/subnet-learn. Supplementary Tables S1, S2, Supplementary Figs. S1 and S2 are included in the supplementary information section.
