# Author Correction: Subnetwork representation learning for discovering network biomarkers in predicting lymph node metastasis in early oral cancer

**DOI:** 10.1038/s41598-021-04481-4

**Published:** 2022-01-05

**Authors:** Minsu Kim, Sangseon Lee, Sangsoo Lim, Doh Young Lee, Sun Kim

**Affiliations:** 1 Computer Science and Mathematics Division, Oak Ridge National Laboratory, Oak Ridge, TN 37831 USA; 2 Institute of Computer Technology, Seoul National University, Seoul, 08826 Korea; 3 Bioinformatics Institute, Seoul National University, Seoul, 08826 Korea; 4 Seoul National University College of Medicine, Seoul, 03080 Korea; 5 Department of Computer Science and Engineering, Seoul National University, Seoul, 08826 Korea; 6 Interdisciplinary Program in Bioinformatics, Seoul National University, Seoul, 08826 Korea; 7 Interdisciplinary Program in Artificial Intelligence, Seoul National University, Seoul, 08826 Korea; 8 AIgenDrug, Co., Ltd, Seoul, Korea

Correction to: *Scientific Reports* 10.1038/s41598-021-03333-5, published online 14 December 2021

In the original version of this Article, Doh Young Lee was omitted as a corresponding author. Correspondence and requests for materials should also be addressed to gedo0212@naver.com.

