# Supplementary material for: Subnetwork representation learning for discovering network biomarkers in predicting lymph node metastasis in early oral cancer
Source: Sci Rep. 2021 Dec 14;11:23992. doi: 10.1038/s41598-021-03333-5 (PMC8671417; doi:10.1038/s41598-021-03333-5)
Supplement: Supplementary file 1 — Supplementary Information 1. [file 41598_2021_3333_MOESM1_ESM.pdf]

Subnetwork representation learning for discovering network biomarkers in predicting lymph node metastasis in early oral cancer

Minsu Kim, Sangseon Lee, Sangsoo Lim, Doh Young Lee, and Sun Kim*

September 29, 2021

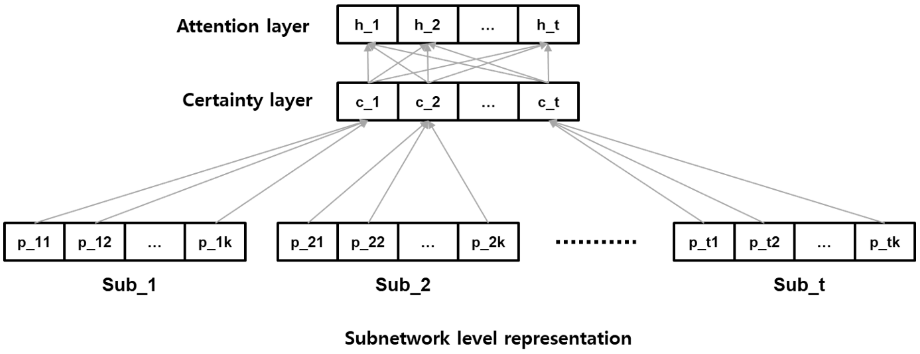

Fig. 1: Attention layer structure

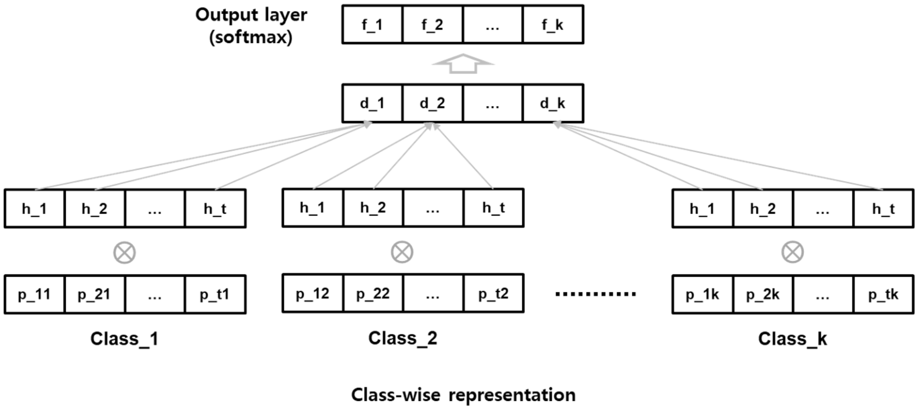

Fig. 2: Decision making process

Table 1: Description of hyperparameters used for the proposed method evaluation

Parameter	Options
Optimization[1]	SGD, RMSprop, AdaGrad, AdaDelta, Adam, AdaMax, and Nadam
Loss[2]	Mean squared error, Mean absolute error, Mean absolute percentage error, Hinge, Mean squared logarithmic error, Logcosh, Squared hinge, Categorical hinge, Categorical crossentropy, Poisson, Sparse categorical crossentropy, Binary crossentropy, Cosine proximity, and Kullback-Leibler divergence
Dropout rate[3]	0.1, 0.2, 0.25, 0.3, 0.5
Split ratio[4]	0.1, 0.2, 0.25, 0.3, 0.5
Feature selection[5]	True and False
Total	4,900 combinations

1 Type of algorithm for optimizing attention layer

2 Type of loss function for optimizing attention layer

3 Dropout rate for each back propagation for optimizing attention layer

4 Validation split ratio of training data for early stopping

5 Whether using feature selection or not

Feature selection refers to the process of filtering features before building a model. For baseline performance, the feature represents each gene while the feature in the proposed method represents each subnetwork. The selection criteria is the classification accuracy produced by the LR model using 3-fold cross-validation (CV). In both cases, sub-average accuracy is filtered out if the feature selection option is set to True.

In summary, the parameter space used in the existing methods (LR, RF, SVM, etc.) is 25,664, and the parameter space used in our proposed method is 4,900.

Table 2: Description of parameters used for the baseline performance evaluation

Parameter	Options
Algorithm[1]	LR, KNN, RF, SVM, and MLP
Kernel[2]	linear, polynomial, RBF, and sigmoid
Size of dimension[3]	Not-reduced, 1, 2, ..., 100
Number of estimator[4]	1, 2, 4, ..., 1024
Number of hidden layers[5]	100, 1000, and 10000
TPM logarization[6]	True and False
Feature selection[7]	True and False
Total	25,664 combinations

1 Type of algorithm used for constructing prediction model

2 Type of kernel used for Kernel Principal Component Analysis (KPCA)

3 Size of dimension used for KPCA

4 Number of estimators in case of using RF algorithm

5 Number of hidden layers in case of using MLP algorithm

6 Whether using logarization or not

7 Whether using feature selection or not
